# Uncomfortable Paresthesia and Dysesthesia Following Tonic Spinal Cord Stimulator Implantation

**DOI:** 10.3390/brainsci15060616

**Published:** 2025-06-07

**Authors:** Zubin Sethi, Tabish Aijaz, Alvaro Ortega-Camacho, Ned F. Nasr, Nebojsa Nick Knezevic

**Affiliations:** 1 Department of Anesthesiology, Advocate Illinois Masonic Medical Center, Chicago, IL 60657, USA; zubin.sethi@aah.org (Z.S.); tabish.aijaz@outlook.com (T.A.); alvarorafaelcamacho@gmail.com (A.O.-C.); ned.nasr@aah.org (N.F.N.); 2 Department of Anesthesiology, University of Illinois, Chicago, IL 60607, USA; 3 Department of Surgery, University of Illinois, Chicago, IL 60607, USA

**Keywords:** neuromodulation, spinal cord stimulation, paresthesia, smoking, explantation

## Abstract

**Background/Objectives**: To determine the incidence of and risk factors for uncomfortable paresthesia and/or dysesthesia after tonic percutaneous spinal cord stimulator implantation. **Methods**: A retrospective analysis was conducted on the prospectively collected data of patients that had permanent percutaneous tonic spinal cord stimulators implanted. Our primary objective was to assess the prevalence of complications over a period of 24 months after the implantation of this device. **Results**: The mean post-implantation follow-up time was 27.3 months. The mean pain score before spinal cord stimulator implantation was 8.05, which was reduced to 3.6 after 24 months. The most common complications in our study sample were the need for revision and the development of unpleasant paresthesia, which were reported by 34.95% and 27.86% of patients, respectively. There was no association between paresthesia and age, sex, or body mass index (BMI). The only risk factor of statistical significance was current tobacco use (*p* = 0.001). **Conclusions**: The development of uncomfortable paresthesia after SCS is associated with considerable morbidity, particularly the explantation of SCS, despite adequate pain relief. Focusing on strategies, such as appropriate waveform selection, might reduce the incidence of uncomfortable paresthesia requiring revision or explantation. Current tobacco use appears to be a significant risk factor for the development of unpleasant paresthesia/dysesthesia when compared with non-tobacco and former tobacco users after tonic percutaneous spinal cord stimulator implantation.

## 1. Introduction

Since gaining FDA approval in 1989, neuromodulation through spinal cord stimulation has been utilized increasingly and has been recommended in the treatment of many chronic pain conditions, including complex regional pain syndrome (CRPS), persistent spinal pain syndrome (PSPS), and chronic pain syndrome with neuropathic pain [1,2]. Spinal cord stimulation initially involved applying pulsed electrical energy in the intrathecal space and has since evolved to implanting leads in the epidural space. This approach to neurostimulation originated from Melzack and Wall’s gate control theory, which proposed that stimulating touch and vibration fibers can interrupt ascending pain signals at the spinal cord, effectively “closing the gate” on pain. Ultimately, the sensation of pain involves an intricate interplay between peripheral nociceptors, second-order neurons in the spinal cord, and projection neurons transmitting signals to the brainstem [3,4,5].

The indications of intractable back pain, PSPS, neuralgia, and CRPS have collectively accounted for 83.6% of all clinical trials of spinal cord stimulators [1]. The target population primarily hones in on those who have failed conservative management, including 6 weeks of physical therapy, cognitive behavioral therapy (CBT), and medical management (NSAIDS, acetaminophen, muscle relaxants, etc.). While the exact rate of spinal cord stimulator placement is yet to be studied, it is estimated that approximately 50,000 spinal cord stimulators are implanted on an annual basis, and recent data suggests an underestimation of implantation [6,7]. This growth highlights the societal need for a better understanding of the complications of this intervention.

While shown to be efficacious in providing pain relief and reducing the usage of analgesic medications, spinal cord stimulation causes a variety of complications. These complications can be biologic, such as infection; hardware-related, such lead migration or fracture; paresthesia-related discomfort; or simply loss of efficacy [8]. These complications may ultimately lead to explantation. Studies have shown an estimated explantation rate of 30%, with lack or loss of efficacy being the most common reason in 26.6–43.9% of explantations [8,9].

**Figure 1:**
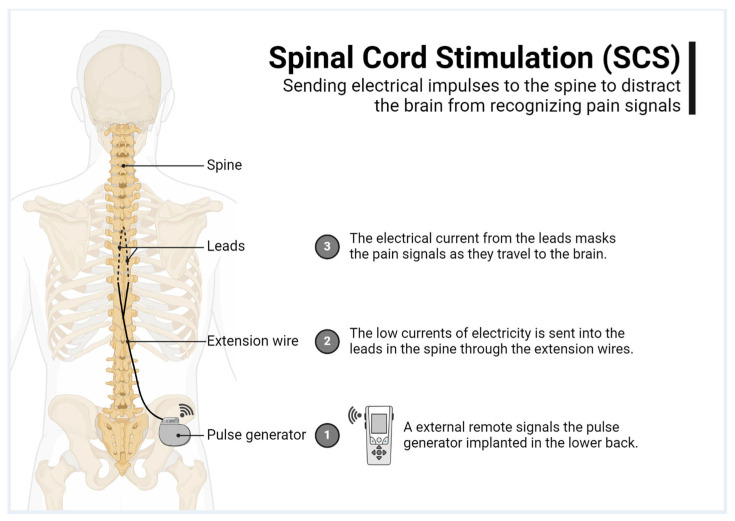
Spinal cord stimulation explained.

Our study primarily aimed to prospectively analyze the complications associated with tonic spinal cord stimulators and identify associated risk factors to better guide the implantation of these devices. A secondary objective of this study was to evaluate the effectiveness of SCS treatment in providing pain relief (Figure 1).

## 2. Materials and Methods

The Advocate Healthcare Institutional Board Review approved this study protocol. An analysis of a prospectively collected database of 118 patients that underwent SCS was performed retrospectively. We included patients with age ≥ 18 years suffering from chronic intractable pain of the trunk and/or limbs, including unilateral or bilateral pain associated with PSPS or intractable low back and leg pain, for a duration of ≥6 months, that was inadequately responsive (or refractory) to supervised conservative care (this includes physical therapy for at least 6 weeks, NSAIDs, acetaminophen, muscle relaxants, and CBT therapy), with a baseline Numerical Rating Scale (NRS) score of six or above for two weeks prior to implantation. Further inclusion criteria included not having undergone surgery of the spine for back or leg pain, Neck Disability Index ≥ 35%, and an Oswestry Disability Index score ≥ 30%. Essentially, these patients make up the target population for spinal cord stimulation.

We excluded patients whose back pain was due to spinal instability (defined as >2 mm translation on radiographic imaging), visceral causes (e.g., endometriosis or fibroids), vascular causes (e.g., aortic aneurysm), spinal infection (e.g., osteomyelitis), inflammation or damage to the spinal cord (e.g., arachnoiditis or syringomyelia), tumor or spinal metastasis, the presence of widespread pain (e.g., fibromyalgia), or pain in other area(s) not intended to be treated in this study (e.g., neck pain, shoulder pain). Further exclusion criteria included seronegative spondyloarthropathy (e.g., rheumatoid, lupus, and psoriatic); neurologic deficits (e.g., foot drop); prior lumbar spine surgery or sacroiliac joint fusion; bedbound status; a morphine-equivalent daily dose of more than 50 MEQ at any time in the prior 90 days; regular intake of systemic steroids (except inhaled steroids used to treat asthma); known allergic reaction(s) to implanted materials; severe scoliotic deformity (>11 degrees in thoracic or lumbar spine); a history of (or existing) intrathecal drug pump; previous experience with neuromodulation devices (including a failed trail); a BMI > 40; enrollment or intention to participate in another clinical drug/device study or registry that might interfere with the results of this study; the presence of other anatomic or comorbid conditions; other medical, social, or psychological conditions that could limit the patient’s ability to participate in the study or to comply with follow-up requirements; failure of a psychological evaluation (performed by independent mental health providers, utilizing variety of scales/questionnaires to both rule out psychological disorders that could influence response to treatment and to comply with health insurance requirements for treatment); evidence of untreated mental illness or substance abuse; the demonstration of two or more Waddell’s signs; current litigation for back pain/injury (or currently receiving worker’s compensation); and current pregnancy/nursing or those planning for pregnancy during follow-up period (female patients of child-bearing age must have a negative pregnancy test done within 7 days prior to enrollment).

Patients underwent MRI within 90 days prior to implantation to evaluate disease severity and identify appropriate location(s) for lead placement. Patients underwent the procedure at Advocate Illinois Masonic Medical Center (New York, NY, , USA), following a stepwise SCS protocol that included a one-week trial period during which all patients received percutaneous leads. All SCSs were implanted by pain fellowship-trained physicians under fluoroscopic guidance to ensure the accuracy and appropriateness of the implant location. To determine further eligibility, the Numerical Rating Scale (NRS) was used during this trial period to assess pain intensity from 0 (“no pain”) to 10 (“worst possible pain”). Those who experienced a ≥ 50% improvement during the trial period received permanent SCS implantation and treatment. Three SCS manufactures were utilized, including Abbot, Medtronic, and Boston Scientific. These manufacturers all produce similar tonic spinal cord stimulators, which emit a frequency anywhere from 10 to 150 Hz and an amplitude up to 25 mA, adjusted based on the individual, to provide constant stimulation to induce paresthesia. For tonic stimulation, these manufacturers produce a near-identical product, while they significantly differ for SCSs that go beyond tonic, such as those that produce burst, utilize amplitude, or utilize other pulse characteristics in diverse ways to deliver diverse therapies. The permanent SCSs were subsequently implanted by experienced, pain fellowship-trained physicians per protocol. Following permanent system implantation, the stimulators were activated and programmed either during the post-operative recovery period or at an office visit, in accordance with the physician’s standard operating procedures. The devices were programmed by trained representatives from their respective device company.

**Figure 2:**
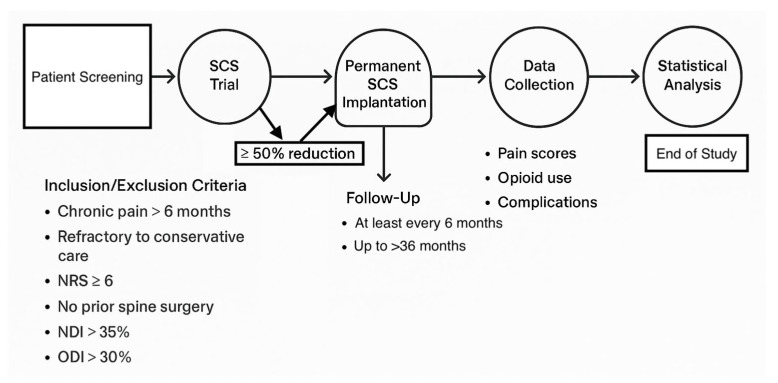
Study timeline.

The follow-up visits were conducted at a 6-month interval for two years after the implantation of permanent SCS. We collected demographic parameters such as age, sex, occupation, smoking status, date of permanent SCS procedure, indication for the procedure, and lead tip location. Pain scores, opioid consumption, and a summary of the complications were recorded at each follow-up visit. The Numeric Rating Scale (NRS) was used to measure the pain score, which is the most common measure used for pain score in clinical practice. Complications were also recorded at each visit, which included infection, seroma, lead migration, paresthesia/dysesthesia, and explantation (Figure 2).

For the purpose of statistical analysis, patients were classified based on age, sex, race, occupation, indication for implantation, region of implantation, and smoking status. The mean and standard deviation of the NRS were calculated at each follow-up visit in all groups and compared by one-way analysis of variance (ANOVA). Further analysis of different complications was performed using the chi-square test. We established an alpha level of 0.05 for all statistical tests. We used SPSS 22.0 software (IBM Corp. Armonk, New York, NY, USA) to perform the analyses.

## 3. Results

### 3.1. Demographics

**Figure 3:**
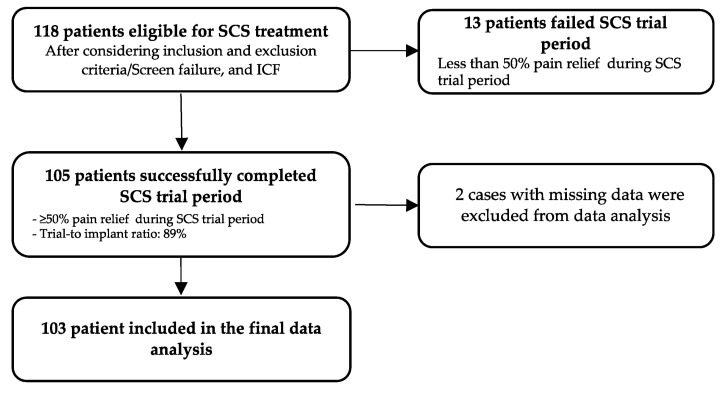
Selection of the sample.

After reviewing the data and adjusting for missing information, 103 patients were included in the final data analysis. Our patient population’s mean age was 55.48 years, with a predominantly female (67%) distribution. The most common indication for implantation in all groups was persistent spinal pain syndrome type 2 (PSPS, 37.86%), followed by low back pain (LBP, 34%). The SCS leads were predominantly placed in the lower thoracic region or below the T10 level (77.67%). After the trial period, 105 received the permanent SCS with a trial-to-implant ratio of 89% (Figure 3). The mean post-implantation follow-up time was 27.3 months. See the population characteristics in Table 1 below.

### 3.2. Effectiveness of SCS Therapy

**Figure 4:**
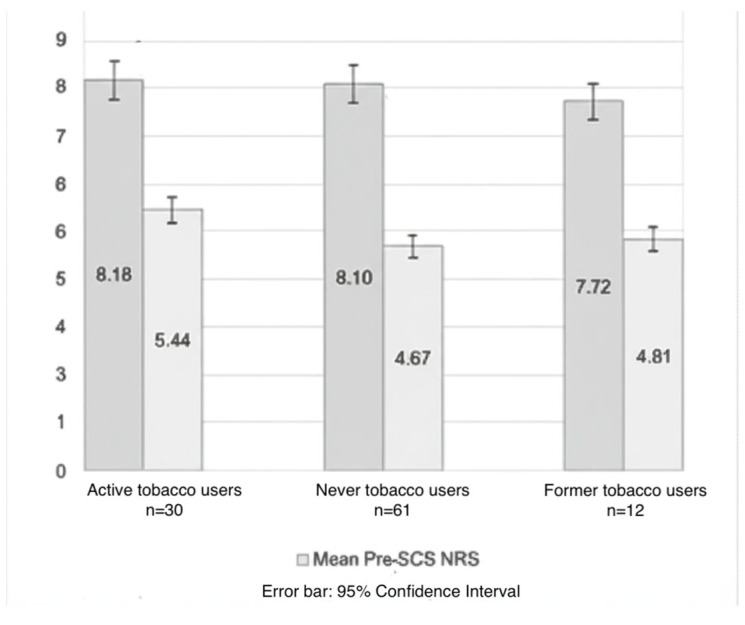
NRS score by smoking status.

The average baseline pain score in all patients before the SCS implantation was 8.09 ± 1.4. The average reduction in pain scores was 60%, which was clinically and statistically significant across all groups (3.10 ± 2.76 reduction on the NRS, *p* < 0.001). The baseline score was higher in the tobacco-user population than non-tobacco users and former smokers, with no statistically significant differences between groups (*p* = 0.65; Figure 4). During the follow-up period, active tobacco users and former tobacco users had a lower than average mean NRS reduction of 2.74 ± 2.3 and 2.90 ± 3.9, respectively, when compared to a 3.33 ± 2.67 mean NRS reduction in never tobacco users (3); however, no statistical significance was observed between the groups (*p* = 0.60).

### 3.3. Summary of Complications

**Figure 5:**
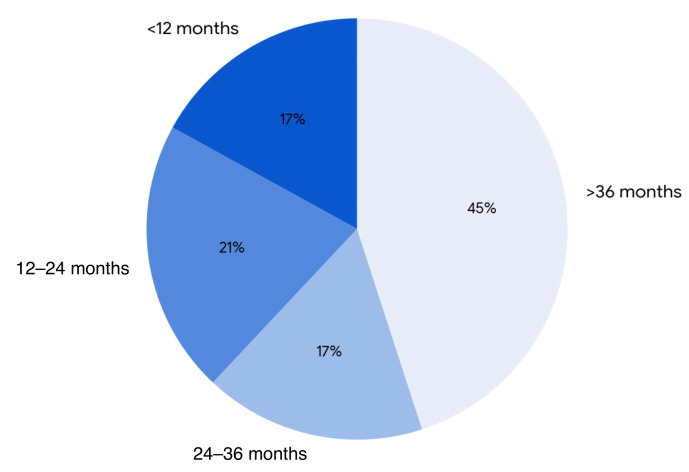
Percentage of complications by time of onset.

On average, postoperative follow-up was conducted at 27.3 months post-implantation. Excluding the need for routine reprogramming, 45% of complications in all groups presented within the first 12 months of follow-up (*p* = 0.022) (Figure 5).

The most common complications were the need for revision, uncomfortable paresthesia/dysesthesia, the need for explantation, lead migration, infection, and loss of effect, respectively. After identifying these complications, we retrospectively analyzed the data to identify specific risk factors. When we classified the patients that received permanent implants according to their smoking status, 30 patients were classified as active tobacco users, 61 as nonsmokers, and 12 patients as former smokers.

#### 3.3.1. Need for Revision

During the follow-up period, 34.95% of patients required revision of their SCS. We found a statistically significant correlation between current tobacco use and cases that required revision (*p* = 0.03). Smoking was the only risk factor in our study that was significantly associated with the need for revision.

#### 3.3.2. Uncomfortable Paresthesia/Dysesthesia

Patients that reported having uncomfortable paresthesia/dysesthesia at the generator site, in the dermatomes to be covered by the SCS, or in areas not intended to be targeted were considered for this category. In our study, 27% of the participants reported having unwanted paresthesia and dysesthesia side effects. We found a statistically significant difference between current tobacco use and cases of uncomfortable paresthesia/dysesthesia in patients that received the permanent SCS (*p* = 0.04) [Table 2]. Smoking was the only risk factor in our study that was significantly associated with the risk of developing uncomfortable paresthesia. Paresthesia was not associated with worsening pain, but it was associated with an increased likelihood of explantation of SCS. There was not a statistically significant difference in pain scores between people who felt paresthesia compared to those without paresthesia. For those who experienced uncomfortable paresthesia/dysesthesia, the mean baseline NRS score was 7.94, and the mean NRS score at final follow up was 5.17, resulting in an average change of −2.77. For all patients in our study, the mean baseline NRS score was 8.09, and the mean NRS score at final follow up was 4.99, resulting in an average change of −3.10.

#### 3.3.3. Explantation and Failure of Treatment

In our study, 12.62% of patients underwent explantation of their device [Table 2]. About 3.8% of the study participants reported the loss of pain coverage, without hardware malfunction or other causes that could explain the failure, and proceeded to undergo explantation.

#### 3.3.4. Lead Migration

Lead migration (LM) is defined as a displacement of the lead from its original desired location by more than one spinal cord segment, and it is typically identified after losing coverage to the targeted dermatome and usually confirmed with x-ray or fluoroscopic examination; 8.73% of the patients in our study presented with LM [Table 2].

#### 3.3.5. Infection/Seroma

During the follow-up period, 5.82% of the study participants developed superficial infections predominantly at the incision site [Table 2]. There were no deep infections reported, and only one case of seroma. These cases were successfully managed with antibiotic therapy or explantation of the device.

## 4. Discussion

The aim of our study was to prospectively analyze the complications associated with tonic spinal cord stimulators and identify associated risk factors to better guide the implantation of these devices. Our results show that the most common complications associated with tonic spinal cord stimulator treatment were the need for revision and uncomfortable paresthesia/dysesthesia, accounting for 51.46% of complications in total.

Our revision rate of 34.95% [Table 2] is consistent with a 2018 retrospective study of 100 patients, where 34 underwent revision surgery [10]. However, a more recent retrospective study published in 2023 analyzing over 1000 patients revealed a notably lower rate of revision (9.5%) [11]. The leading cause for revision in that study was lead migration/misplacement (32%), which is notably higher than the 8.73% of our patients who experienced lead migration. The stark contrast between our study and this 2023 study could be attributed to the advancement of this technology, including the addition of “anchors” to keep the device in place, as well as the proficiency of the specialists performing the implantation. Device revision is a costly procedure that negatively impacts patient satisfaction, and the success of device revision has been shown to decrease as the number of revisions increases [12]. In our study, the only significant risk factor for the incidence of device revision was smoking. Given the scarcity of the literature investigating the effect of tobacco on revision rate, our clinically and statistically significant results may guide future practice and research.

Our second most common complication was the feeling of uncomfortable paresthesia/dysesthesia, which developed in 27.86% of patients. This is consistent with a in 15-year single-center retrospective study, where paresthesia-related side effects and limitations accounted for 26.6% of explantations [8]. This begs the question of whether traditional low frequency tonic spinal cord stimulators, which were implanted in our study, should be largely replaced by the newer paresthesia-free stimulators, such as those utilizing high frequency or bursts of stimulation.

Spinal cord stimulators vary based on the frequency of the waveform they emit. The original mechanism, known as tonic, emits a frequency of 10 to 150 Hz, an amplitude up to 25 mA, and provides constant stimulation to the area in question, providing paresthesia to the area in pain, replacing the sensation of pain [13,14]. While some patients found this paresthesia to be a comfortable feeling, many have found this stimulated paresthesia to be uncomfortable [14,15,16]. Over time, newer techniques for spinal cord stimulation have been developed, which include high-frequency spinal cord stimulation and burst spinal cord stimulation. Both techniques, respectively, through providing constant, high frequency, or a burst of five pulses at a lower frequency, provide stimulation at a level below the threshold of perceiving paresthesia and thus provide paresthesia-free pain relief [15]. A 2016 multicenter randomized control trial of 198 patients over 2 years revealed that 11.3% of patients with traditional tonic spinal cord stimulators experienced unpleasant paresthesia, compared to 0% of those with high-frequency spinal cord stimulators [17]. This is consistent with a 2017 review that claimed that burst and high-frequency stimulators may provide superior pain relief without this unpleasant tingling paresthesia associated with tonic stimulators [18]. It is important to note that, at the time of our study, many of the newer devices described above were either not available or in the investigational stage. Interestingly, despite the advent of newer technology, some patients still prefer tonic stimulation, further highlighting the importance of our study. Our authors posit this to be due to certain patients getting a sense that the treatment is taking effect upon feeling the paresthesia.

Tonic stimulators are still a major component in the current landscape of SCS treatment, and some emphasize their continued importance in managing chronic pain [14]. A 2022 Survey of the active members of the Spine Intervention Society (SIS) and American Society of Regional Anesthesia (ASRA) revealed a relatively similar usage rate between tonic, burst, and high-frequency (10 kHz) stimulators [19]. A retrospective study in the UK analyzing the medical records of nearly 1200 patients from April 2008 to December 2018 revealed that tonic stimulators were predominantly used over high-frequency (10 khz) and burst stimulators, respectively, representing 57% of the patients. The authors of that study found a statistically significant increased risk of explantation of tonic stimulators compared to both high-frequency (10 khz) and burst stimulators [20]. Interestingly, the FDA has deemed high-frequency (10 khz) stimulators superior to tonic stimulators by comparing data documenting leg pain reduction [15]. Further, the SUNBURST (Success Using Neuromodulation With BURST) Study, a 2018 prospective, randomized controlled trial, revealed that 17% of patients experienced paresthesia with burst stimulation compared to 92% in the tonic stimulation group, and that 69% of patients preferred burst stimulators over tonic stimulators [21]. There appears to be a great depth of literature detailing the superiority of these newer spinal cord stimulators in comparison to the tonic technique.

Additionally, our findings suggest that the development of unpleasant paresthesia/dysesthesia after tonic percutaneous spinal cord stimulator implantation is associated with smoking tobacco. This was the only risk factor in our study that was significantly associated with our two most common complications: the need for revision and uncomfortable paresthesia. Those patients who reported current or former exposure to tobacco had 7.2 times the risk of developing a complication during the SCS follow-up period (95% CI: 2.7–19, *p* < 0.0001). Our results are comparable to prior literature, which concluded that tobacco use increases the risk of SCS postoperative complications, such as the need for revision, lead migration, and SCS failure [22,23,24,25]. The exact mechanism by which smoking increases the risk of uncomfortable paresthesia is not yet clear. The literature has shown that nicotine affects multiple receptors in the brain, including opioid, cholinergic or serotonergic, noradrenergic, and dopaminergic receptors [26,27]. Chronic nicotine use causes dysregulation of these neurotransmitters and their associated pathways in a manner that makes these patients more sensitive to pain [28,29,30,31,32]. While spinal cord stimulation has been shown to restore autonomic dysregulation, its impact on patients who continue to smoke is not yet known [33]. Another proposed mechanism is that smoking produces oxidative stress substances, such as acrolein, that upregulate certain receptors involved in pain perception, resulting in hyperalgesia that may last up to 1 week following smoking cessation [34]. Much of the current literature regarding smoking and neuromodulation is centered around the perioperative, intraoperative, and immediate post-operative timelines, which supports the need for future studies on the long-term impact of smoking on neuromodulation [35,36].

A secondary objective of this study was to evaluate the effectiveness of SCS treatment in providing pain relief. Overall, all patients in our cohort, regardless of age, sex, occupation, smoking status, date of permanent SCS procedure, indication for the procedure, or lead tip location, saw a statistically and clinically significant reduction in pain, as measured by NRS score. This is notable to mention, as it contrasts with a retrospective cohort study published in 2018 that concluded that current smokers had statistically significant lower reductions in NRS score compared to both nonsmokers and former smokers. They also found a statistically significant increase in median opioid consumption in current smokers compared to nonsmokers, as opioid intake was 2.4 times higher (*p* = 0.004) in smokers than in lifelong nonsmokers [23]. We agree with the authors of that study that further study and level I evidence is needed to change guidelines of clinical practice, and that patients should continue to be counseled and educated regarding smoking cessation. Our authors have previously raised the question of avoiding implantation in smokers or possibly instituting a longer trial period. Our study builds on previous research by highlighting the importance of understanding patient demographics when it comes to SCS therapy and showing that it remains plausible for smokers to have a statistically and clinically significant reduction in NRS scores. We hope that our study will lead to new insights into guiding a different neuromodulation algorithm for smokers, such as defining an optimal period of abstinence and correlating this to the amount that someone has smoked during their lifetime (pack-years, etc.) [37,38].

Regarding the future of this therapy and guidance for future studies, the topic of tobacco use should be further explored, as we mentioned above. If we take into consideration the study by Mekhail et al., which suggested a negative association between smoking and SCS efficacy in chronic spine-related pain patients, along with our finding that current smokers did experience statistically significant pain reduction, we should institute a protocol focused on cessation, but not one that would bar smokers from receiving this therapy. If patients choose to continue smoking, this should be discussed in the informed consent process, specifying that they are less likely to experience the intended effect and are more likely to experience complications. Additionally, paresthesia-free stimulators, such as those utilizing high frequency, may be more appropriate than tonic stimulators for smokers, and there is a societal need future studies exploring this topic.

One of our main limitations is that this is a retrospective study with a small sample size; therefore, our results may not be sufficient to obtain reasonable statistics. Our study’s association cannot be confirmed as causal in nature due to the study design. Despite adjusting for confounding variables, there is residual bias caused by uncontrolled confounding variables (such as neuropathy caused by diseases unrelated to the indication of SCS therapy and other unspecified comorbidities). Additionally, despite having a mean follow-up time of 27.3 months, there is heterogeneity in the data regarding follow-up time and smoking quit-time. Another limitation is that we could not assess the functional status of patients after the implantation of SCS, which is an important aspect to consider while using SCS. Future research should study the comparison of the different waveforms in a subgroup of patients with a history of smoking and associated long-term effects.

## 5. Conclusions

Our study identifies modifiable risk factors with clear areas of potential improvement that could help reduce SCS failure and explantation rates. The development of paresthesia after SCS is a commonly encountered complication following SCS implantation that is associated with considerable morbidity, despite not being associated with worsening pain. Very few studies have examined the relationship between patient characteristics and long-term complications of SCS. Lastly, smoking appears to be associated with the development of unpleasant paresthesia/dysesthesia following SCS implantation. 

## Figures and Tables

**Table 1:** Population characteristics.

Population Characteristics (n = 103)	
Age (years)	
(Mean, SD)	55.48 ± 13.08
Sex	
Male	34 (33%)
Female	69 (67%)
Occupation	
Working	36 (34.95%)
Disability	23 (22.33%)
Modified Duty	6 (5.83%)
Retired	23 (22.33%)
Unemployed	15 (14.56%)
Indication of SCS	
PSPS	39 (37.86%)
CRPS	14 (13.60%)
LBP	35 (34%)
Other	15 (14.54%)
SCS implantation region	
Cervical	9 (8.74%)
Upper thoracic	4 (3.88%)
Lower thoracic	80 (77.67%)
Other	10 (9.71%)
Months of follow up	
(Median, IQR)	20 (28)

SD = standard deviation, SCS = spinal cord stimulator, PSPS = persistent spinal pain syndrome, CRPS = complex regional pain syndrome, and LBP = low back pain. IQR = interquartile range.

**Table 2:** Complication outcomes stratified by smoking status.

Complications	Active Tobacco Users (n = 30)	Never Tobacco Users (n = 61)	Former Tobacco Users (n = 12)	Total (n =103)	*p* Value
**Infection**	1 (3.33%)	2 (3.27%)	1 (8.33%)	6 (5.82%)	0.69
**Lead Migration**	5 (16.67%)	3 (4.91%)	1 (8.33%)	9 (8.73%)	0.17
**Revision**	16 (53.33%)	16 (26.22%)	4 (33.33%)	36 (34.95%)	0.03
**Explantation**	6 (20%)	2 (3.27%)	5 (41.67%)	13 (12.62%)	0.09
**Dysesthesia/Paresthesia**	10 (33.33%)	4 (6.55%)	3 (25%)	17 (27.86%)	0.04
**Loss of Effect**	2 (6.66%)	0	2 (16.67%)	4 (3.88%)	0.01

## Data Availability

The original contributions presented in this study are included in the article. Further inquiries can be directed to the corresponding author.

## Abbreviations

The following abbreviations are used in this manuscript:

SCS	Spinal cord stimulator
CRPS	Complex regional pain syndrome
PSPS	Persistent spinal pain syndrome
NRS	Numerical Rating Scale
LBP	Low back pain
